# Characterization of Polyphenol and Volatile Fractions of Californian-Style Black Olives and Innovative Application of E-nose for Acrylamide Determination

**DOI:** 10.3390/foods10122973

**Published:** 2021-12-02

**Authors:** Elísabet Martín-Tornero, Ramiro Sánchez, Jesús Lozano, Manuel Martínez, Patricia Arroyo, Daniel Martín-Vertedor

**Affiliations:** 1 Department of Agricultural and Forestry Engineering, School of Agrarian Engineering, Universidad de Extremadura, 06007 Badajoz, Spain; emartintornero@gmail.com (E.M.-T.); mmcano@unex.es (M.M.); 2 Technological Institute of Food and Agriculture CICYTEX-INTAEX, Junta of Extremadura, Avda, Adolfo Suárez s/n, 06007 Badajoz, Spain; ramiro.sanchez@juntaex.es; 3 Perception and Intelligent Systems Research Group, Universidad de Extremadura, 06006 Badajoz, Spain; jesuslozano@unex.es (J.L.); parroyoz@unex.es (P.A.); 4 Research Institute of Agricultural Resources INURA. Avda de la Investigación s/n, Campus Universitario, 06006 Badajoz, Spain

**Keywords:** table olives, sterilization treatments, volatile compounds, acrylamide, electronic nose

## Abstract

Californian-style black olives require a sterilization treatment that produces a carcinogenic contaminant, acrylamide. Thus, this compound was evaluated in two different olive cultivars using an electronic nose (E-nose). The sterilization intensity had a significant influence on the final phenol concentrations, acrylamide content, and volatile compounds. Increasing the sterilization intensity from 10 to 26 min (F0) reduced the phenol content, but it promoted acrylamide synthesis, leading to a wide range of this toxic substance. The Ester and phenol groups of volatile compounds decreased their content when the sterilization treatment increased; however, aldehyde and other volatile compound groups significantly increased their contents according to the thermal treatments. The compounds 4-ethenyl-pyridine, benzaldehyde, and 2,4-dimethyl-hexane are volatile compounds with unpleasant odours and demonstrated a high amount of influence on the differences found after the application of the thermal treatments. The “Manzanilla Cacereña” variety presented the highest amount of phenolic compounds and the lowest acrylamide content. Finally, it was found that acrylamide content is correlated with volatile compounds, which was determined using multiple linear regression analysis (*R^2^* = 0.9994). Furthermore, the aroma of table olives was analysed using an E-nose, and these results combined with Partial Least Square (PLS) were shown to be an accurate method (range to error ratio (RER) >10 and ratio of performance to deviation (RPD) >2.5) for the indirect quantification of this toxic substance.

## 1. Introduction

Olive tree cultivation mainly extends throughout the Mediterranean area, with the commercial uses of olives mainly being the production of oil and table olives. Table olive production exceeded 2.9 million tons during the 2019–2020 season, with the main producers being Spain, Egypt, Turkey, Algeria, Italy, Greece, and Portugal [1,2]. The nutritional relevance of table olives is widely known. Olives are a good source of good monounsaturated fat, vitamin e, polyphenols, and flavonoids (antioxidants with anti-inflammatory benefits).

Californian-style black olive is one of the most important elaboration processes in the table olive sector. These olives are often consumed on pizzas, and canned olives are preserved and are used in other ways. The popularity of Mediterranean cuisine also contributes to sustained consumption. The main variety used in this process is “Hojiblanca”, a variety that is produced in Andalusia, followed by “Manzanilla Cacereña” or “Manzanilla Sevillana” [3,4]. To produce ripe black olives, the olives are harvested when they are green (unripe), and they are then cured through exposure to a series of air oxidation and lye treatments to obtain debittered black olives. The olives are placed in cans and are subjected to a thermal sterilization treatment.

High temperatures above 120 °C promote acrylamide synthesis, a process that seems to be initiated through a chemical reaction such as the Maillard reaction, which is related to carbohydrates and amino acids (asparagine) [4,5,6]. However, the synthesis of this toxic substance has also recently been related to fat-rich table olives [7]. The intensity of the thermal sterilization process applied in each industry produces different acrylamide concentrations in oxidized olives ranging from 30 to 1050 ng g^−1^ [4,6]. This wide range in acrylamide content is mainly due to the fact that each industry applies a different thermal sterilization treatment. Thus, industries should control sterilization treatments to apply the minimum amount of heat necessary to achieve a microbiologically stable product with the least amount of acrylamide.

This chemical molecule is classified as toxic and carcinogenic and has demonstrated adverse health effects [5,6,7,8]. Furthermore, the European Food Safety Authority considers this kind of olives as a potential source of acrylamide since these table olives contain higher levels of the compound compared to other foods such as chips, bread, cookies, coffee, chocolate, or cocoa [9,10]. For this reason, knowledge of the acrylamide content in table olives is important for human health. A European recommendation [11] indicates that authorities must control the acrylamide level in olives stored in brine solutions on an industrial scale. In this sense, several results in the literature apply different mitigation strategies to reduce acrylamide levels during the industrial elaboration process of Californian-style black [4,5,12,13] and green olives [14].

Acrylamide and volatile compound quantification require long-term extraction and purification methods [6,15]. Moreover, analyses are carried out using expensive equipment, such as with mass spectrometry detectors that are similar to those used for gas [4,16] or liquid chromatography [6,12,17]. They turn out to be expensive, complex, and time-consuming techniques that require trained personnel.

Electronic devices are able to evaluate the volatile organic compounds that are responsible for the characteristic odour patterns of the food. These devices commonly called electronic noses (E-nose) and consist of an array of gas sensors and a data acquisition block, and the application of data processing techniques has proven to be a useful tool for the evaluation of olives. However, electronic nose devices have not been used to predict the acrylamide content in foods or Californian-style black olives as of yet. In the literature, we were only able to find other devices with voltammetric biosensors for detecting this toxic substance in foods [18,19]. Recently, studies have been carried out using electronic noses to predict the quality of the oil to be processed from the measurements obtained in freshly harvested olives [20] and to evaluate the odours produced in anomalous fermentations of Spanish-style green olives [15]. The variation of the volatile organic compounds generated in food after thermal treatments has also been studied to monitor the evaluation of the quality of the oil used for frying [21], to identify the olfactory profile of rapeseed oil as a function of heating time [22], or in the sensory evaluation of Californian-style black olive sterilization treatments [23].

Thus, this work aimed to assess the chemical composition in terms of phenol profile, antioxidant activity, acrylamide content, and volatile compounds in Californian-style black olives (“Manzanilla Cacereña” and “Hojiblanca” varieties) that have been submitted to different thermal treatments (10–26 min at 121 ± 3 °C) to find out whether the E-nose can be used to determine acrylamide content.

## 2. Materials and Methods

### 2.1. Experimental Design

Two olives varieties (“Hojiblanca” and “Manzanilla Cacereña”) were harvested at the green stage of maturation during the 2020/21 season in the “Vegas Bajas del Guadiana” area. The olives were stored for 4 months with acetic acid (1.5% *v*/*v*) and salt (8% *p*/*v*) in industrial tanks in triplicate. The olive comprised Californian-style black olives, and the olives were packed into cans with 2% NaCl and 0.015% ferrous gluconate [13].

The cans were sterilized at a specific temperature (121 ± 3 °C) with the different equivalent treatment durations (in minutes) required to reduce the initial microbial load. Cans were submitted to five sterilization treatments using the accumulated lethality for reducing the microbial population, which was calculated as F0 values by integrating the time–temperature profile recorded for each treatment that was studied: (i) F0 = 10 min (T1), (ii) F0 = 14 min (T2), (iii) F0 = 18 min (T3), (iv) F0 = 22 min (T4), and (v) F0 = 26 min (T5). All of the cans were stored at room temperature. The experiments were performed four times.

### 2.2. Chemical and Reagents

For Californian-style black olive elaboration, an acetic acid solution was purchased from Panreac Applichem^®^ (Darmstadt, Germany), ferrous gluconate and sodium chloride were supplied by Sigma-Aldrich (St. Louis, MO, USA), and calcium chloride was obtained by Tetra Chemicals (Helsingborg, Sweden).

The following analytical standards were used for phenolic profile analysis: hydroxytyrosol, procyanidin B1 (PB1), procyanidin B2 (PB2), apigenin-7-O-glucoside, oleuropein, and verbascoside were supplied by Extrasynthése (Genay, France), and tyrosol, vanillic acid, epicatechin, luteolin-7-O-glucosidem and p-coumaric acid were supplied by Sigma-Aldrich Chemie (Steinheim, Germany). P.A. grade formic acid was purchased from PANREAC (Barcelona, Spain), and sodium fluoride was supplied by Sigma-Aldrich Chemie (Steinheim, Germany). HPLC grade acetonitrile and methanol were provided from Fisher chemical (Loughborough, UK). For the antioxidant activity, DPPH (2,2-diphenyl-1-picrylhydrazyl) and 6-hydroxy-2,5,7,8-tetramethyl-chroman-2-carboxylic acid (Trolox) were supplied by Alfa-Aesar (Kandel, Germany) and Sigma-Aldrich (Steinheim, Germany), respectively.

Acrylamide and 2,3,3-D3-acrylamide were acquired from Fluka (Buchs SG, Switzerland) and Cambridge Isotope Laboratories (Andover, MA, USA), respectively. Analytical grade methanol was obtained from Merck (Darmstadt, Germany).

A nylon syringe filter was purchased from FILTER-LAB (Barcelona, Spain). Isolute Multimode (300 mg, 6 mL) and ENV+ (200 mg, 3 mL) Solid Phase Extraction (SPE) columns were obtained from IST (Hengoed, Mid-Glamorgan, UK). Nylon and nitrocellulose syringe filters (0.45 μm) were purchased from Tracer Analytical Technologies (Madrid, Spain). Water was purified with an Elix/Milli-Q water purification system (Millipore, Bedford, MA, USA). For volatile compound analysis, stableflex polydimethylsiloxane/divinylbenzene (PDMS/DVB) fibre was acquired from Supelco. The DB WAXETR capillary column (60 m × 0.25 mm; DI: 0.25 mm) was purchased from Agilent. For the electronic device (E-nose), the commercial sensor array of the oxide semiconductor (MOX) was procured from different manufacturers: (i) Bosch BME680: temperature (°C), pressure (hPa), humidity (% RH), and gas measurement (Ω); (ii) Sensirion SGP30: eCO_2_ (ppm), TVOC (ppb), H_2_ (2), and ethanol; (iii) ScioSense CCS811: eCO_2_ (ppm), TVOC (ppb), and sensor resistance (Ω); and iv) ScioSense iAQ-Core: eCO_2_ (ppm), TVOC (ppb), and sensor resistance (Ω).

### 2.3. Analyses

Different analyses were carried out on Californian-style black olives subjected to thermal treatments. Specifically, phenol chromatographic analysis, antioxidant activity, acrylamide content, volatile compound, and E-nose measurements were performed.

#### 2.3.1. HPLC Analysis of the Phenolic Profile of the Olives

The phenolic extraction and characterization of the phenolic profile were performed according to the methodology described by Cabrera-Bañegil et al. [24] via chromatographic separation. A 2 g sample of table olives was crushed, mixed with a solution of 2 mM NaF in 10 mL of methanol, and homogenized. It was placed in an ultrasonic bath (P-Selecta, mod 513) for 30 min. The extracts were centrifuged at 1677× *g* at 4 °C for 10 min. Finally, the supernatant extracts were filtered through a 0.22 mm nylon syringe filter (FILTER-LAB) before injection into the HPLC system [8,24,25]. Samples were analysed on an Agilent 1100 series HPLC system (Hewlett-Packard, Waldbronn, Germany) equipped with a diode array detector (DAD) and a fluorescence detector (FLD). The analytical column that was employed was a Phenomenex Gemini-NX C18 column (Phenomenex, Torrance, CA, USA), (150 mm × 4.6 mm, 3 μm). The column temperature was set at 40 °C. The injection volume was 10 μL, and the flow rate was 1 mL min^−1^. The mobile phases were 0.1% (*v*/*v*) formic acid in water (eluent A) and 0.1% (*v*/*v*) formic acid in acetonitrile (eluent B). The gradient used was as follows: 0–1 min, 3% B in isocratic mode; 1–30 min, linear gradient from 3% to 35% B; 30–33 min, linear gradient from 35% to 50% B; 33–34 min, linear gradient from 50% to 100%; and 34–50 min, 100% B in isocratic mode. The gradient was then returned to 3% eluent B, and this composition was held for 3 min to re-equilibrate the column. Quercetin and oleuropein were monitored by DAD and were quantified at 255 nm; the benzoic acids were quantified at 280 nm; the cinnamic acids were quantified at 320 nm; flavones and quercetin-3-rutin were quantified at 350 nm; and anthocyanins were quantified at 515 nm. Fluorescence detection at 275/315 nm was used for the analysis of hydroxytyrosol, tyrosol, PB1, catechin, PB2, and epicatechin

#### 2.3.2. DPPH Antioxidant Activity

The phenolic extract obtained from the olive matrix explained in Section 2.3.1 was used to carry out the DPPH (2,2-diphenyl-1-picrylhydrazyl) free radical method. An amount of 300 µL of the phenol extract sample was mixed with 2.7 mL of DPPH methanolic solution. The corresponding mixture was kept in darkness for 1 h, and the absorbance was then measured at 517 nm against a blank (MeOH) in a J.P Selecta, S.A spectrophotometer (Madrid, Spain). The results were expressed as Trolox equivalent values (mg Trolox·100 mL^–1^ extract).

#### 2.3.3. Acrylamide Determination

Acrylamide determination was carried out following the method described by Pérez-Nevado et al. [6] and that had been validated by Fernández et al. [26]. An amount of 2 g of olives was crushed, homogenized, and shaken with 10 mL of Milli-Q water for 60 min. Afterwards, the sample was centrifuged for 30 min at 4 °C at 1677 g. The aqueous phase was filtered through a 0.45 µm nylon syringe filter and was cleaned through a Telos PCX (200 mg/3 mL) solid-phase extraction cartridge. The cartridge was conditioned with 4 mL of methanol followed by 4 mL of Milli-Q water, and 3 mL of the sample was injected into the column and eluted with 3 mL of Milli-Q water. The eluate was introduced in another Telos PRP cartridge (60 mg/3 mL), which had been conditioned in the same way and eluted with 3 mL of Milli-Q water. The quantification of the acrylamide content was carried out by the standard addition method using a standard acrylamide solution (50–150 ng mL^−1^).

Samples were analysed on an Agilent 1290 Infinity II liquid chromatograph (Agilent Technologies, Palo Alto, CA, USA) equipped with a degasser, quaternary pump, column oven, and autosampler, and the Chemstation software was used for instrument control, data acquisition, and data analysis. The analytical column used was a Zorbax Eclipse XDB-C18 column (150 mm × 2.1 mm, 3.5 μm) (Agilent technologies), the temperature of which was set at 30 °C. The mobile phase was composed of 95% solvent A (0.1% formic acid in Milli-Q water) and 5% solvent B (0.1% of formic acid in methanol). The flow rate was 0.25 mL/min, and the injection volume was 3 µL.

Detection was performed with a mass spectrometer Agilent Technologies 6460 triple quadrupole equipped with an electrospray ion source operating in positive ion mode. The ion source parameters were set as follows: gas temperature: 340 °C, gas flow: 12 L h^−1^, nebuliser: 40 psi, sheath gas temp: 400 °C, sheath gas flow: 12 L h^−1^, capillary voltage: +2.5 kV, nozzle voltage: 300 V, and delta EMV: 300. The fragmentary voltage was 50 V, and the collision energy was 9 V and 20 V, respectively.

#### 2.3.4. Volatile Compound Analysis

**Figure 1:**
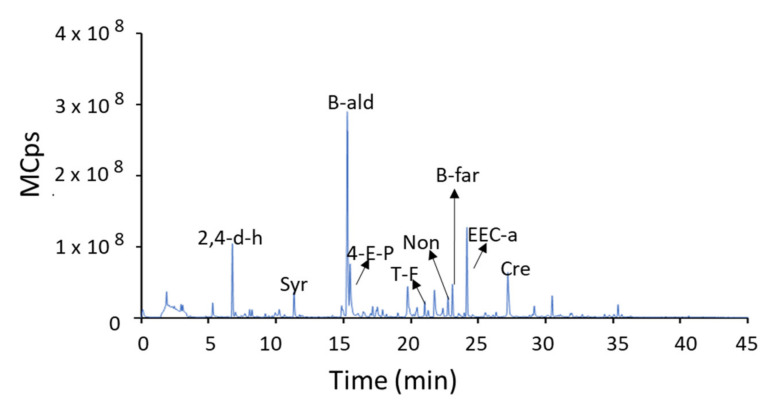
Representative chromatogram of the aromatic profile of thermal sterilized California-style black olives. Syr: styrene; 4-E-P: 4-ethenyl-pyridine; t-F: trans-farnesol; B-ald: benzaldehyde; Non: nonanal; EEC-a: ethyl ester cyclohexanecarboxylic acid; Cre: creosol; 2,4-d-h: 2,4-dimethyl-hexane; b-far: beta-farnesene.

A paste was obtained from the previously deboned and homogenized black olives. An aliquot of 2.0 g of paste was mixed with 7.0 mL of 30% NaCl (*w*/*v*) in a 15 mL glass vial. The volatile compounds were analysed with a Bruker Scion 456-GC triple quadrupole gas chromatograph following a procedure reported in the literature [27]. They were sampled from the headspace at 40 °C for 15 min using an SPME with a polydimethylsiloxane/divinylbenzene (PDMS/DVB) StableFlex fiber (65 μm, Supelco). After SPME, desorption was carried out in the injection port of the gas chromatograph at 250 °C for 15 min. The components were separated using a VF-5MS capillary column (30 m × 0.25 mm; ID: 0.25 mm). The different volatile compounds were identified by comparison with the NIST 2.0 MS library. A representative chromatogram is shown in Figure 1.

#### 2.3.5. E-nose Determination

The E-nose device that was used was small in size (39 mm × 33 mm), portable, low-energy and could be connected to a smartphone via Bluetooth protocol. It was designed by the Research Group on Perception and Intelligent Systems at the University of Extremadura. The E-nose consists of four gas sensor chips with integrated metal oxide (MOX) sensors: BME680, SGP30, CCS811, and iAQ-Core. The microcontroller read the values detected by the sensors, formatted them, and sent them to an external smart device via Bluetooth. All of the sensors, except BME680, include intelligent algorithms to process the raw signals to output TVOC (Total Volatile Organic Compounds) and equivalent CO_2_ (eCO_2_) prediction values. Additionally, SGP30 provides raw signals for H_2_ and ethanol. The BME680 also includes temperature, relative humidity, and atmospheric pressure sensors.

This type of sensor matrix has been successfully used for the evaluation of trichloroanisole (TCA) in cork stoppers [28], air quality measurement [29], or the discrimination of anomalous fermentation defects in table olives [15]. The odour profile measurements of the olive and brine samples were carried out following the recommendations of the International Olive Council [30] for the olive tasting panel.

Samples of olives with brine were placed in the tasting glasses, covered with a clock glass, and placed in a heating block at 25 °C. Each data acquisition cycle consisted of two parts after an initial period of sensor stabilization. The first part corresponded to a 30 s desorption period, during which the sensors were placed in an empty cup. In the second part of the cycle, the volatile absorption achieved by the table olives was analysed at one-second intervals for 60 s. Five measurements were taken for each table olive sample.

### 2.4. Data Analysis

Two-way ANOVA was performed to determine the significant differences between olive varieties and the sterilization treatments on the different chemical compounds being studied. One-way ANOVA and Tukey’s test were conducted when the interaction effect was not statistically significant at a level of *p* < 0.05. SPSS 18.0 software (SPSS Inc., Chicago, IL, USA) was used to perform the analysis. The results were expressed as mean values ± standard deviation.

### 2.5. Chemometric Analysis

Chemometric analysis was performed using Matlab 2016b version 9.1 (Math-Works, Natick, MA, USA) with the PLS-toolbox 8.2.1 (Eigenvector Research, Inc., Wenatchee, WA, USA).

Multiple Linear Regression (MLR) was used to identify the relationship between the percentage of the volatile compounds and the acrylamide concentration. The coefficient of determination (R^2^) was applied to evaluate the performance of the applied model.

The partial least squares (PLS) method [31] was used to build a model for the quantification of acrylamide from the E-nose data. The data set was split into a calibration set (70% of the samples), which was used to calibrate and cross-validate the models, and a validation set (30%), which was only used to test the robustness and accuracy of the developed models. The samples were divided randomly between the two sets. The optimal number of latent variables (LV) was optimized based on the calculation of the minimum root mean square error.

The model performance was estimated using the following statistic parameters: the coefficient of determination for calibration (R^2^_cal_), cross-validation (R^2^_CV_), and prediction (R^2^_P_) (Equation (1)); the root mean square error for calibration (RMSEC), cross-validation (RMSECV), and prediction (RMSEP) (Equation (2)); and the ratio of performance to deviation (RPD) (Equation (3)) and the range to error ratio (RER) (Equation (4)).

(1) $$R^{2}=1-\frac{\sum {\left(y_{\mathrm{pred}}-y_{\exp}\right)}^{2}}{\sum {\left(y_{\mathrm{pred}}-y_{\mathrm{mean}}\right)}^{2}}$$

(2) $$\mathrm{RMSE}=\sqrt{\sum \frac{{\left(y_{\mathrm{pred}}-y_{\exp}\right)}^{2}}{N}}$$

(3) $$\mathrm{RPD}=\frac{\mathrm{SD}}{\mathrm{RMSEP}}$$

(4) $$\mathrm{RER}=\frac{\left(y_{\max}-y_{\min}\right)}{\mathrm{RMSEP}}$$

where y_exp_ is the experimental value; y_pred_ is the corresponding value obtained for the calibration (R^2^_cal_ and RMSEC), cross-validation (R^2^_CV_ and RMSECV), and prediction (R^2^_P_ and RMSEP); N is the number of samples; and SD is the standard deviation of the experimental values.

The PLS model can be regarded as an acceptable model if it has a low number of PCs, low RMSE values, high R^2^, an RPD value greater than 2, and a low gap between the two sets (i.e., calibration and validation) [32]. RER values above 10 indicate that the models were adequately identified [33].

## 3. Results

### 3.1. Effect of Thermal Treatments on the Chemical Properties

The chemical properties studied in Californian-style table olives submitted to different thermal treatments are reported in Table 1. A two-way ANOVA analysis was computed to assess the effect of the olive varieties and sterilization treatments. A significant interaction was found in almost all of the variables that were studied, so data are shown for each olive variety and thermal treatment studied. Thus, the phenolic compounds profile and antioxidant activity were performed after each thermal treatment and at the same time as when the acrylamide formation and the volatile compounds were being analysed.

#### 3.1.1. Effect of the Sterilization Treatments on the Antioxidant Properties

The phenolic profiles of the table olives allowed for the separation and identification of 10 phenols. Qualitative differences in these compounds were found after the application of different sterilization treatments on the studied varieties. Regardless of the intensity of the thermal treatment applied, hydroxytyrosol was the main phenolic compound recorded in the table olives that were studied, followed by tyrosol and oleuropein. The other minor phenolic compounds were PB1, p-coumaric, verbascoside, apigenin-7-O-glucoside, vanillic acid, luteolin-7-O-glucoside, and epicatechin.

The application of different intensities of thermal treatments in the present study had a significant influence on the final phenol concentrations. Thus, the increase of the sterilization intensity from F0 values of 10 to 26 min reduced the phenol contents in Californian-style black olives in both of the studied varieties. In fact, when the sterilization treatment was more aggressive, the total content of phenolic compounds decreased by more than 50% in both varieties. Although almost all of the phenolic compounds significantly decreased their initial content; the phenols that were the most sensitive to sterilization treatments were verbascoside and p-coumaric, the contents of which decreased by more than 80%, while the least sensitive were epicatechin and vanillic acid, which decreased by 20%. Hydroxytyrosol, oleuropein, p-coumaric, or the sum of the phenol profiles (Σ phenols) that were analysed decreased significantly with increasing amount of sterilization treatments; however, other phenols such as tyrosol, PB1, or vanillic acid were more or less affected depending on the intensity of the thermal treatment.

Furthermore, differences related to phenolic compounds were found when comparing different Californian-style black olive varieties. The highest concentrations were found in the “Manzanilla Cacereña” variety in all of the sterilization treatments that were studied. The total phenol profiles (Σ phenols) were more than 20% higher in “Manzanilla Cacereña” than they were in “Hojiblanca” regardless of the thermal treatment applied. The phenols with the least varietal influence were vanillic acid and epicatechin, especially when the sterilization treatments were greater.

Concerning the antioxidant profile of the two varieties considered herein, the results are depicted in Table 1. The DPPH antioxidant method showed significant differences between the thermal treatment applied and the table olive varieties studied. The preliminary results suggested that the strongest antioxidant activity was found when the thermal treatments were less aggressive in both varieties. The variety that showed the highest anti-radical activity was “Manzanilla Cacereña”.

#### 3.1.2. Effect of Thermal Treatments on Acrylamide Content

The acrylamide levels that were analysed by HPLC-MS-QQQ in olives submitted to different sterilization treatments are shown in Table 1. According to the results, there is a significant interaction effect (*p* ≤ 0.05) between the olive cultivar and the thermal sterilization treatments. Thus, the results are presented individually for each olive variety that was studied and the thermal treatments that were applied.

The sterilization treatments that were used in the last step of the elaboration process of the Californian-style black olive had a huge influence on the acrylamide synthesis. This led to a wide range of concentrations of this toxic substance (105.4 to 446.1 ng·g^−1^) depending on the applied time-period treatment and olive variety used. In fact, when the applied thermal intensity was too aggressive (T5), the acrylamide content increased by more than 70% with respect to treatment T1. Thus, with the gentler thermal treatments are used (T1–T3), the increase in acrylamide content is not as high.

In addition, the olive variety also was determined to influence acrylamide levels. The variety with the highest acrylamide content was “Hojiblanca”, while the lowest level of this toxic substance was observed in the “Manzanilla Cacereña” variety. In both varieties, the acrylamide content increased with the cumulative sterilization treatments. In fact, the “Hojiblanca” variety presented higher acrylamide levels in a range of 14 to 23% depending on the thermal treatment applied.

Finally, the correlation between the level of acrylamide and phenols was carried out by taking into account the high influence of phenols on the formation of this toxic substance. Depending on the phenolic compound that was studied, the Pearson correlation varied its significance level. A negative correlation was observed for most of the phenols analysed and for the sum of the total phenols (Σ phenols) with an acrylamide concentration. The compounds with the highest correlation were hydroxytyrosol (*R^2^* = 0.90), tysosol (*R^2^* = 0.73), PB1 (*R^2^* = 0.78), oleuropein (*R^2^* = 0.82), luteolin-7-O-glucoside (*R^2^* = 0.91), apigenin-7-O (*R^2^* = 0.86), verbascoside (*R^2^* = 0.77), *p*-coumaric (*R^2^* = 0.93), and Σ phenols (*R^2^* = 0.88).

#### 3.1.3. Effect of Thermal Treatments on the Volatile Compounds

The volatile compounds of Californian-style black olive from both varieties submitted to different thermal treatments were classified into six types according to chemical group (Table 2). The group of volatile compounds with the highest representation in this elaboration process was aldehydes, followed by esters, phenols, aromatics, other compounds, and alcohols.

The thermal treatments caused a variation in the volatile compound profile of the treated olives. Aromatics, aldehydes, and other volatile compound groups increased their content according to the thermal treatment. The alcohol group was under-represented in this type of olives and did not present a clear trend regarding the effect on the thermal treatments that were applied. On the other hand, the volatile compound groups comprising esters and phenols decreased their content when the sterilization treatment increased. Moreover, the volatile compounds in each group stabilized their contents with the most aggressive sterilization treatments (T4–T5).

**Figure 2:**
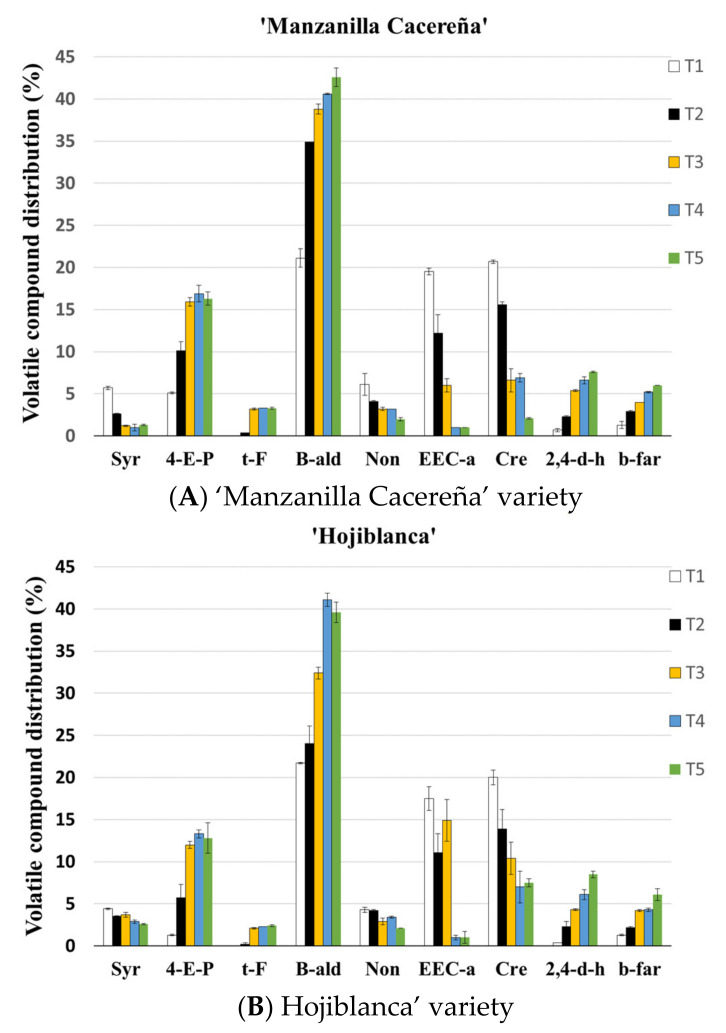
Relative contents of volatile compounds (mean% (*n* = 4)) obtained from Californian-style black olives from “Manzanilla Cacereña”(**A**) and “Hojiblanca”(**B**) varieties and submitted to five different sterilization treatments: T1: F0 = 10 min; T2: F0 = 14 min; T3 F0 = 18 min; T4: F0 = 22 min; and T5: F0 = 26 min. Syr: styrene; 4-E-P: 4-ethenyl-pyridine; t-F: trans-farnesol; B-ald: benzaldehyde; Non: nonanal; EEC-a: ethyl ester cyclohexanecarboxylic acid; Cre: creosol; 2,4-d-h: 2,4-dimethyl-hexane; b-far: beta-farnesene.

Furthermore, although almost 50 volatile compounds were identified, the major constituents in the olive matrix are shown in Figure 2 The unpleasant odour aromas such as 4-ethenyl-pyridine (aromatics), benzaldehyde (aldehydes), and 2,4-dimethyl-hexane (others), which had a high influence after the application of the thermal treatments, increased in content. Moreover, other fruity aromas, such as ethyl ester cyclohexanecarboxylic acid (esters) and creosol (phenols), decreased their content [23,34,35].

The behaviour of the volatile compounds determined to be present in the volatile profiles of both of the olive varieties that were studied followed a similar synthesis and degradation pattern. However, some groups of volatile compounds were synthesized more in one variety than they were in the other. Such is the case of the aromatic group in which the “Manzanilla Cacereña” variety presented higher values. This is due to the higher content of the 4-ethenyl-pyridine. The rest of the volatile compounds present similar values when soft and more aggressive thermal treatments were applied (T1 and T5).

### 3.2. Relationship between Volatile Compounds and Acrylamide Content

As seen in Section 3.1.3, the volatile compound profile varied with the thermal treatment, just as the concentration of acrylamide increased with the increasing thermal treatments.

To determine whether certain volatile compounds could be correlated with the acrylamide content to some extent, a correlation study with the individual volatile compounds was carried out. The results showed that many variables were positively or negatively correlated with acrylamide, demonstrating R^2^ values above 0.6. Acrylamide content waspositively correlated with the percentage of 2,4-dimethyl-hexane, (Rr^2^ = 0.87), 2-heptyn-1-ol (R^2^ = 0.88), benzaldehyde (R^2^ = 0.81), 4-ethenyl-pyridine (R^2^ = 0.67), octanal (R^2^ = 0.88), trans-farnesol (R^2^ = 0.81), and beta-farnesene (R^2^ = 0.84). Moreover, a negative correlation was found with ethyl ester hexanoic acid (R^2^ = 0.61), nonanal (R^2^ = 0.71), ethyl ester cyclohexanecarboxylic acid (R^2^ = 0.72), and creosol (R^2^ = 0.89).

**Figure 3:**
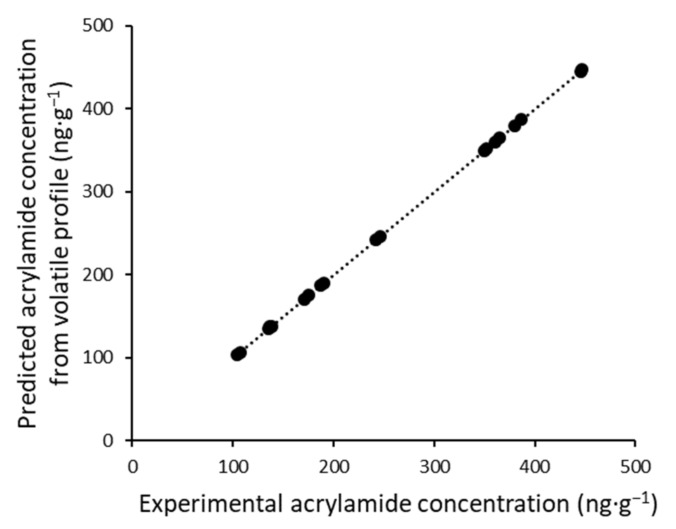
Predicted acrylamide concentration by volatile profile vs. experimental value.

Based on these results, we studied the possibility of finding a regression model between these two types of variables. An MLR model was built between the relative contents of all of the volatile compounds that were found and the experimental acrylamide content obtained by the reference method. The result is shown in Figure 3, where we can observe the good correlation (r^2^ = 0.994) between the acrylamide concentration quantified by the traditional HPLC method and the predicted concentration obtained by the model.

### 3.3. Acrylamide Quantification by Using the E-nose

From the results of the previous section, it could be said that it is possible to predict the acrylamide content from the volatile compound profile. However, it is still a very expensive and time-consuming technique. Therefore, the use of the E-nose for the indirect determination of acrylamide was studied.

E-nose signals were obtained from the measurements of Californian-style black olives submitted to different thermal treatments. Before performing the quantitative model, feature extraction was conducted in order to characterize the sensor response curves. The feature that was selected was the maximum signal value minus the minimum signal value multiplied by 100 and subtracted by 1. These data were used to perform a partial least squares (PLS) model to evaluate the relationship between the E-nose measurements and the experimental values of the acrylamide concentration.

The PLS model was optimized using the calibration set. The number of LVs used in the calibration was selected using the leave-one-out cross-validation by the lowest RMSECV and the higher R^2^cal. The best number of LVs was 4. The resulting PLS model was then tested with the validation set. Based on this model, the calculation of the merit figures was performed, and the results are shown in Table 3. The model shows high linearity with R^2^cal, which has a value of 0.85, and R^2^cv and R^2^pred, which show values of 0.79 and 0.78, respectively. The root mean square error in both the cross-validation and validation were considered low. Moreover, from this table, it was also observed that it is possible to determine the acrylamide content with relatively high accuracy (RER and RPD above 10 and 2.5, respectively).

**Figure 4:**
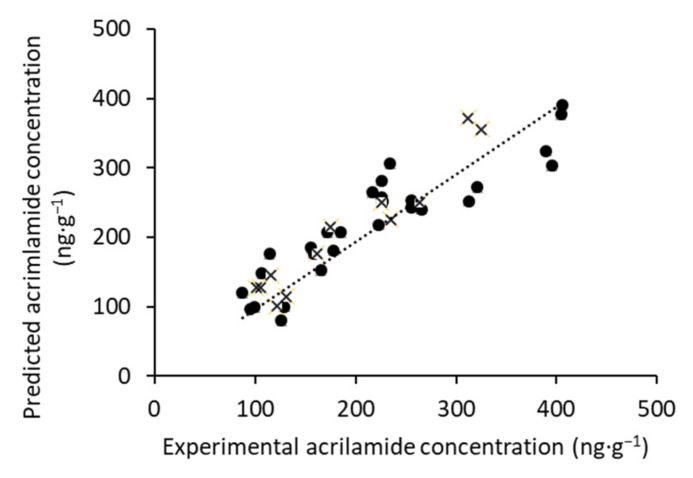
Correlation plot of calibration (•) and prediction (×) sets for the determination of acrylamide concentration.

These results are corroborated by the graph of predicted values obtained by the PLS model versus the experimental values obtained by the reference method. As shown in Figure 4, there is a solid correlation between predicted and measured values over the calibration and validation sets. As such, the results of this PLS model are efficient and point out the fact that this technology can be used to estimate the acrylamide content in Californian-style black olives with no sample preparation.

## 4. Discussion

The chemical composition of California-style table olives varies according to the intensity of the thermal treatments used. Phenols are known to be thermosensitive [6,36], which is confirmed in the present study. The application of different intensities of thermal treatments in Californian-style black olives resulted in a significant reduction of these compounds. Moreover, it should be noted that the phenolic profile of this type of olive elaboration is quite poor in terms of the number of identified and quantified phenolic compounds. In other types of table olive production, a much broader phenolic profile appears. For instance, unfermented table olives from different varieties were studied by Franco et al. [37] and showed a phenolic profile of more than 24 phenols. Lodolini et al. [8] studied the phenolic profile of different table olive elaboration processes and showed that the highest contents of these compounds were found in the natural style. Pistarino et al. [38] found lower hydroxytyrosol concentrations in fermented table olives of the “Taggiasca” variety, whereas similar tyrosol concentrations were reported. Furthermore, all the elaboration processes where lye and thermal treatments are applied provoke a decrease in phenolic compound content [24].

Despite this, thermal sterilization is necessary for this type of table olives to ensure food safety by the inactivation of the bacteria spores. However, the acrylamide concentration was affected by the intensity of the thermal treatments. It should be noted that an F0 value lower than 18 min resulted in acrylamide synthesis below 250 ng g^−1^. On the contrary, sterilization treatments longer than 22 min clearly exceed this proposed limit concentration, which could have a negative effect on people’s health [5,8,9]. Therefore, it should be noted that the industries producing this type of olive must regulate the intensity of the thermal treatments that are applied to sterilize the olives in order to increase the quality of the final product. Acrylamide generation in table olives is more pronounced in Californian-style black ripe olives. This elaboration process involves oxidation with air, treatment with sodium hydroxide, washing phases, the addition of ferrous gluconate, and thermal sterilization (121 °C during 20–30 min). In this sense, different strategies have been proposed to mitigate the presence of this toxic substance during its industrial processing [13,14]. These strategies are based on the reduction of the precursor of this pollutant. The different phases in which some improvements have been proposed are (a) table olive maturation stage; (b) length of the storage period; (c) types and times of washing treatments before oxidation; (d) table olive presentation formats; (e) NaCl addition; and (f) CaCl_2_ addition. Thus, the minimum levels of acrylamide in table olives marketed in Europe should be set at values closer to 300 µg/kg, thus guaranteeing the health of consumers of table olives at an international level. In this way, the application of mitigation measures and good practices at the industrial level is promoted to meet the levels stipulated by the regulation. The intake of Californian-style table olives with high acrylamide content could cause serious health problems.

In our results, it is shown that those olives with a high phenolic compound content were those that presented the least amount of acrylamide. Different studies indicate that phenols can inhibit acrylamide synthesis and that sometimes, the results that are obtained by different research groups are contradictory. In fact, the olives with the highest hydroxytyrosol content presented higher acrylamide reduction [39]. However, other phenolic compounds could also act against acrylamide syntheses such as hydroxytyrosol, tyrosol, and oleuropein [40], which also appears in oxidized olives. In addition, certain natural additives such as blanched garlic, oregano, or rosemary showed a significant reduction in acrylamide [39].

Pérez Nevado et al. [6] found that the presence of antioxidant compounds in olives is directly proportional to the mitigation of acrylamide levels when the olives are both fresh and after having undergone some thermal treatment. As a result of this observation, it the addition of antioxidant compounds to the olive, in form of phenolic compounds, to check if there is any contaminant mitigation has been studied [12]. After inoculation with these bioactive compounds, these researchers showed that acrylamide formation decreased by 35% for the olive leaf extracts and by 54% for the mixture of the extract and the hydroxytyrosol.

Regarding the volatile compounds, Californian-style black olives have a characteristic odour of a cooked sensory note [15,23,41] that is mainly provoked by the thermal treatment that was used to stabilize these types of olives. Following IOC regulations, the results obtained by these researchers can classify table olives that have been submitted to different sterilization treatments into different sensory categories after considering the predominantly perceived defect (PPD) and intensity attributes. Thus, olives that are subjected to a more aggressive sterilization treatment are classified as low quality, leading to there being a greater cooking effect sensation in the olives. Therefore, the results show that the odorous compounds are directly related to the intensity of the applied thermal treatments. The behaviour of the aroma is similar to that of phenols, as these compounds are strongly influenced by the heat applied. Some volatile compound groups increase considerably in table olives with the application of different thermal sterilization treatments, while other different groups decrease. For example, the content of phenols, alcohols, and esters decreased as the F0 increased. However, the aldehyde content decreased, probably due to oxidative processes [42], with the aldehydes being the main products of lipid oxidation and amino acid degradation [43].

Some the volatile compounds such as benzaldehyde, 4-ethenylpyridine or 2,4-dimethylhexane are related to the cooking effect and an unpleasant odour. López-López et al. [27] showed that the volatile compounds with an unpleasant odour are synthesized with the application of temperature higher than 121 °C. In the literature, it is widely described that thermal sterilization treatments cause this characteristic cooking effect that is seen in olives and that this effect is caused by long exposure to heat in the olive cans. In addition, some researchers [13] have described that this cooking effect increases with the applied thermal intensity. Therefore, it is once again of interest, at an industrial level, to regulate the intensity of thermal treatments to obtain olives with a fruitier aromatic profile and less unpleasant aromas.

The olive aroma provided by increased thermal treatments may correlate with increased acrylamide content in both varieties. Several volatile compounds were positively or negatively correlated, with an r^2^ > 0.6, with the acrylamide content. Moreover, an MLR model showed that it would be possible to determine the acrylamide content through the volatile profile.

Therefore, as this linear correlation has been found, the next step was to evaluate the volatile compounds using an electronic nose to indirectly determine the acrylamide content. In this case, a PLS was performed to establish a standard model that served as an indirect method for the quantification of this toxic substance. A linear relationship was established between the measurements predicted by the PLS model and the experimental acrylamide content. Moreover, good quality parameters were obtained in the developed model. Taking the obtained results into account, we can state that it could be possible to predict the acrylamide concentration using the electronic nose.

In the literature, several works establish direct and indirect relationships between other devices and acrylamide content, but this studied represented the first time that an electronic nose was used for this purpose. PLS multivariate analysis has previously been used in the literature to indirectly predict acrylamide content produced during coffee roasting from proton magnetic resonance (NMR) spectra that had already been recorded for other coffee control purposes. In the NMR spectrum, acrylamide is not directly quantifiable, so these researchers established a correlation between the reference value and the corresponding NMR spectrum using a partial least squares (PLS) regression [44]. Furthermore, Martín-Vertedor et al. [41] established a direct linear model between acrylamide content and potentiometric electronic tongue to monitor the content of this toxic substance in Californian-style black olive. Therefore, the use of electronic devices for acrylamide detection is of interest since industries will be able to determine the content of this toxic substance quickly, efficiently, and reliably.

## 5. Conclusions

Phenols, acrylamide, and volatile compound concentration depends on the intensity of the sterilization treatments and on the table olive varieties used as Californian-style black olives. Furthermore, phenolic compounds can have a positive effect on the formation of acrylamide. In fact, olives with a high phenolic compound content had a lower content of this toxic substance. The thermal treatments that were applied caused a change in the phenol and aromatic profile of the olives, which is directly related to the acrylamide content. Thus, the aromatic profile of the olives was also detected by the E-nose, establishing a relationship with the acrylamide content. In this way, this portable device is faster than the conventional method and could be interesting for industries to control the amount of this toxic substance before bringing it to the market.

## Figures and Tables

**Table 1:** Chemical composition of Californian-style black olives (“Manzanilla Cacereña” and “Hojiblanca”) submitted to different thermal sterilization treatments. Results are expressed as mean ± SD of three sample replicates. For the same cultivar, different lowercase letters mean a statistically significant difference between thermal treatment (one-way ANOVA followed by Tukey’s test, *p* < 0.05).

**‘Manzanilla Cacereña’**					
	***T1***	***T2***	**T3**	**T4**	**T5**
***Phenolic profile (mg·100 g^−1^)***					
Hydroxytyrosol	1226.6 ± 69.8 ^e B^	1004.5 ± 32.8 ^d B^	920.1 ± 14.5 ^c B^	680.5 ± 25.8 ^b B^	551.8 ± 26.2 ^a B^
Tyrosol	312.1 ± 6.4 ^d B^	205.4 ± 10.5 ^c B^	196.6 ± 16.1 ^c B^	143.7 ± 5.7 ^b B^	127.0 ± 6.3 ^a B^
PB1	41.4 ± 3.5 ^d B^	40.6 ± 3.6 ^d B^	27.5 ± 5.5 ^c B^	19.1 ± 1.6 ^b NS^	15.5 ± 1.0 ^a B^
Vanillic acid	6.8 ± 0.5 ^b B^	3.8 ± 0.2 ^a NS^	4.1 ± 0.2 ^a NS^	3.7 ± 0.1 ^a NS^	3.7 ± 0.1 ^a NS^
Epicatechin	4.3 ± 0.1 ^c NS^	4.8 ± 0.7 ^d B^	3.9 ± 0.5 ^b NS^	3.8 ± 0.5 ^b NS^	3.2 ± 0.1 ^a NS^
Oleuropein	239.4 ± 10.9 ^e B^	206.8 ± 4.8 ^d B^	155.0 ± 7.4 ^c B^	125.8 ± 3.4 ^b B^	99.6 ± 1.5 ^a B^
Luteolin-7-O-glucoside	6.1 ± 0.6 ^e B^	4.9 ± 0.1 ^d B^	4.1 ± 0.2 ^c NS^	1.8 ± 0.2 ^b B^	1.4 ± 0.1 ^a NS^
Apigenin-7-O	7.8 ± 0.6 ^c B^	6.1 ± 1.4 ^b B^	6.2 ± 0.2 ^b NS^	2.7 ± 0.2 ^a B^	2.5 ± 0.1 ^a B^
Verbascoside	8.9 ± 1.1 ^d B^	9.6 ± 0.7 ^d B^	7.4 ± 0.3 ^c B^	1.9 ± 0.1 ^b B^	1.3 ± 0.1 ^a B^
p-coumaric	21.6 ± 1.4 ^e B^	18.7 ± 0.5 ^d B^	16.4 ± 0.7 ^c B^	3.4 ± 0.4 ^b B^	2.4 ± 0.1 ^a B^
Σ phenols	1874.9 ± 77.9 ^e B^	1505.2 ± 47.4 ^d B^	1341.2 ± 32.7 ^c B^	986.4 ± 28.6 ^b B^	808.5 ± 30.4 ^a B^
***Antioxidant properties (mgTrolox·g extrac^−1^)***					
DPPH	2.7 ± 0.1 ^d B^	2.6 ± 0.2 ^d B^	2.5 ± 0.3 ^c B^	2.2 ± 0.1 ^b B^	1.9 ± 0.1 ^a B^
***Toxic substance (ng·g^−1^)***					
Acrylamide	105.4 ± 3.4 ^a A^	137.7 ± 3.1 ^b A^	188.7 ± 5.2 ^c A^	312.4 ± 6.0 ^d A^	383.5 ± 8.9 ^e A^
**‘Hojiblanca’**					
	***T1***	***T2***	**T3**	**T4**	**T5**
***Phenolic profile (mg·100 g^−1^)***					
Hydroxytyrosol	911.2 ± 9.9 ^e A^	839.0 ± 15.6 ^d A^	793.4 ± 14.6 ^c A^	526.4 ± 3.0 ^b A^	425.8 ± 6.0 ^a A^
Tyrosol	194.0 ± 7.8 ^e A^	164.4 ± 5.3 ^d A^	152.2 ± 4.1 ^c A^	104.9 ± 5.2 ^b A^	89.6 ± 3.5 ^a A^
PB1	30.9 ± 1.2 ^d A^	23.3 ± 1.1 ^c A^	20.2 ± 2.4 ^c A^	17.3 ± 2.6 ^b NS^	9.7 ± 0.5 ^a A^
Vanillic acid	5.0 ± 0.1 ^c A^	4.1 ± 0.2 ^b NS^	3.9 ± 0.3 ^b NS^	3.1 ± 0.1 ^a NS^	3.1 ± 0.1 ^a NS^
Epicatechin	3.7 ± 0.3 ^ns NS^	3.2 ± 0.1 ^ns A^	3.9 ± 0.4 ^ns NS^	4.2 ± 0.1 ^ns NS^	2.8 ± 0.6 ^ns NS^
Oleuropein	173.2 ± 9.4 ^e A^	150.8 ± 8.4 ^d A^	141.6 ± 9.8 ^c A^	113.8 ± 3.0 ^b A^	91.9 ± 6.5 ^a A^
Luteolin-7-O-glucoside	5.2 ± 0.2 ^c A^	4.4 ± 0.1 ^b A^	4.3 ± 0.3 ^b NS^	1.3 ± 0.2 ^a A^	1.3 ± 0.1 ^a NS^
Apigenin-7-O	6.1 ± 0.1 ^c A^	5.1 ± 0.2 ^c A^	6.2 ± 0.6 ^c NS^	2.2 ± 0.1 ^b A^	1.2 ± 0.1 ^a A^
Verbascoside	4.6 ± 1.0 ^b A^	5.4 ± 0.2 ^b A^	4.9 ± 0.1 ^b A^	1.2 ± 0.2 ^a A^	1.1 ± 0.1 ^a A^
p-coumaric	16.5 ± 1.1 ^c A^	16.6 ± 0.5 ^c A^	10.7 ± 0.5 ^b A^	2.7 ± 0.2 ^a A^	1.9 ± 0.1 ^a A^
Σ phenols	1350.3 ± 20.6 ^e A^	1216.4 ± 24.7 ^d A^	1141.3 ± 10.6 ^c A^	777.1 ± 9.4 ^b A^	628.5 ± 12.2 ^a A^
***Antioxidant properties (mgTrolox·g extrac^−1^)***					
DPPH	1.0 ± 0.1 ^d A^	1.0 ± 0.1 ^d A^	0.9 ± 0.1 ^c A^	0.8 ± 0.1 ^b A^	0.6 ± 0.1 ^a A^
***Toxic substance (ng·g^−1^)***					
Acrylamide	136.7 ± 4.4 ^a B^	172.9 ± 2.8 ^b B^	244.0 ± 5.8 ^c B^	362.8 ± 4.3 ^d B^	446.1 ± 12.9 ^e B^

F0 = 10 min; T2: F0 = 14 min; T3 F0 = 18 min; T4: F0 = 22 min; and T5: F0 = 26 min; ns or NS means no significant differences.

**Table 2:** Distribution (%) of chemical families of volatile compounds in Californian-style black olives (“Manzanilla Cacereña” and “Hojiblanca”) submitted to sterilization treatments (T1–T5).

	‘Manzanilla Cacereña’					‘Hojiblanca’				
	T1	T2	T3	T4	T5	T1	T2	T3	T4	T5
Aromatics	15.5	18.1	25.5	26.9	27.5	7.6	14.2	18.4	20.8	19.3
Alcohols	3.3	2.0	3.7	4.7	4.8	3.2	4.3	4.6	3.7	4.0
Aldehydes	28.1	40.9	42.8	45	46.7	27.2	32.4	37.2	45.9	44.5
Esters	26.1	15.8	8.7	2.3	2.2	27.9	22.4	15.6	5.1	5.2
Phenols	20.9	15.6	6.6	6.9	2.1	20.6	15.9	11.4	7.0	7.5
Others	6.1	7.6	12.7	14.2	16.7	13.5	10.8	12.8	17.5	19.5

T1: F0 = 10 min; T2: F0 = 14 min; T3 F0 = 18 min; T4: F0 = 22 min; and T5: F0 = 26 min.

**Table 3:** Parameters and figures of merit of the PLS model for the determination of acrylamide through the use of E-nose.

LVs	R^2^_cal_	R^2^_CV_	R^2^_P_	RMSEC	RMSECV	RMSEP	RPD	RER
4	0.85	0.79	0.78	35.24	41.48	37.07	2.63	10.61

R^2^_cal_: coefficient of calibration; R^2^_CV_: coefficient of cross-validation; R^2^_pred_: coefficient of prediction; RMSEC: root mean square error of calibration; RMSECV: root mean square error of cross-validation; RMSEP: root mean square error of prediction; RPD: ratio of performance to deviation; RER: range error ratio.

## Data Availability

All relevant data are included within the manuscript. The raw data are available on request from the authors.
